# Beyond the Dip: Silent and Non-Silent Fano Resonances in Quantum Systems

**DOI:** 10.1021/acsomega.5c11827

**Published:** 2026-01-16

**Authors:** Ali K. Ismael

**Affiliations:** † Physics Department, 4396 Lancaster University, Lancaster LA1 4YB, U.K.; ‡ Department of Physics, College of Education for Pure Science, Tikrit University, Tikrit 34001, Iraq

## Abstract

This study presents a theoretical investigation of quantum interference effects on charge transport in a series of bithiophene-bridged molecular derivatives, each functionalized with terminal thiol anchor groups. Using a combined density functional theory and nonequilibrium Green’s function (DFT-NEGF) approach, we demonstrate that the electrical conductance is primarily governed by the amplitude of the frontier molecular orbitals (FMOs) at the anchoring sites, as predicted by the orbital product rule. Furthermore, we show that introducing pendant atoms (oxygen) onto a carbazole core creates localized electronic states that interfere with the delocalized backbone, generating distinct Fano resonances in the transmission spectrum. Derivative **3**, engineered with two oxygen atoms, exhibits a complex quantum interference landscape featuring a pronounced second Fano resonance and a suppressed, “silent” resonance. Crucially, we reveal that the Fano resonance associated with the highly localized HOMO is extremely sensitive to minute fluctuations in the molecule–electrode binding configuration, rendering it experimentally silent. In contrast, a hypothetical symmetric junction achieves perfect unitary transmission due to quantum interference enforced by spatial symmetry. This work provides a detailed blueprint for designing molecular-scale quantum interference devices and highlights the critical role of structural fluctuations in determining measurable conductance.

## Graphical abstract

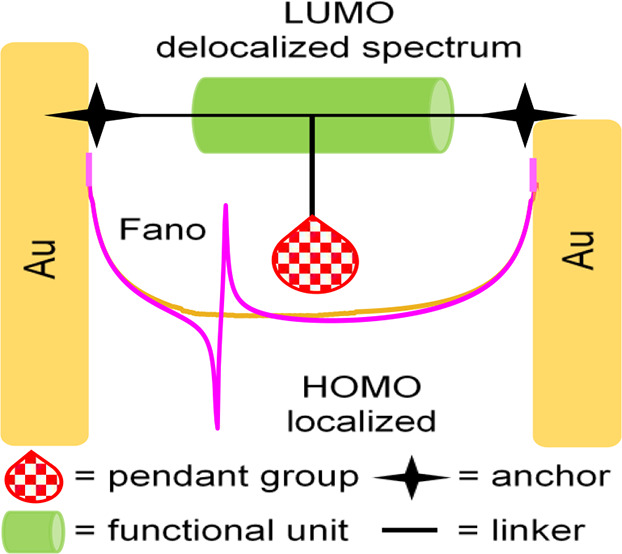

## 1 Introduction

In the burgeoning field of molecular electronics, the exploitation of quantum interference (QI) has emerged as a transformative design principle, enabling unparalleled control over electron transport in nanoscale junctions. This paradigm moves beyond the conventional rules of Ohm’s Law and classical circuit theory, venturing into a realm where the wave nature of electrons dictates device functionality. The core mechanism hinges on the precise tuning of resonant scattering phenomena within a molecular framework. As electron waves traverse a molecule, they probe multiple pathways through its discrete orbital structure. The resultant conductance is then critically determined by the quantum-mechanical phase coherence of these waves, their interplay can yield either constructive interference, creating a transparent resonant state, or destructive interference, leading to a pronounced suppression of current known as an antiresonance.^1–3^ This capacity to engineer electronic transmission through molecular design gives rise to a host of nonclassical tunneling behaviors. These include pronounced negative differential resistance, highly sensitive quantum-based switching, and the ability to achieve exceptional thermoelectric performance by selectively filtering electron energies. Such effects are fundamentally irreplicable in classical electronics, which cannot access this phase-coherent control. Thus, quantum interference provides a powerful toolkit for conceptually innovating electronic device architecture. It allows for the bottom-up creation of components from logic gates and amplifiers to highly efficient energy converters whose properties are intrinsically defined by their quantum mechanical structure, marking a significant leap toward the next frontier of miniaturized and efficient quantum enabled technologies.^4–10^

The investigation of resonant phenomena provides a fundamental framework for understanding quantum transport at the nanoscale. A cornerstone of this understanding is the Breit–Wigner formula,^11^ which describes idealized symmetric resonances. This formalism has been extensively analyzed through the lens of quantum interference, particularly by examining how constructive quantum interference (CQI) can lead to resonant transmission peaks, while destructive quantum interference (DQI) creates pronounced antiresonances or transmission dips. A particularly intriguing manifestation of such interference is the Fano resonance phenomenon. Unlike the symmetric line-shape of a Breit–Wigner resonance, a Fano resonance is characterized by a distinctly asymmetric, sharp profile. This unique shape arises from the quantum mechanical interaction between a discrete, localized energy state (such as a molecular orbital) and a broad continuum of states. In the context of molecular electronics, this continuum is supplied by the electronic bands of the metallic electrodes, which inject electrons into the molecular bridge over a continuous range of energies.^12–15^ This phenomenon is not exclusive to electronic systems, it has been observed across a diverse spectrum of wave scattering systems. Notable examples include electron transport through quantum dots and molecular junctions, as well as light propagation in engineered photonic crystals and plasmonic devices. The universal nature of the Fano effect underscores its importance in nanoscale physics. Despite its prevalence and fundamental significance, the practical application and, crucially, the control of Fano resonances in molecular-scale systems remain a formidable challenge.^16–18^

The primary difficulty lies in the precise engineering of the delicate interference condition between the discrete state and the continuum. This requires atomic-level control over the molecular structure, its coupling to the electrodes, and the external electrostatic environment—a level of precision that is often beyond current experimental capabilities. Consequently, harnessing the sharp, highly sensitive properties of Fano resonances for applications in switching, sensing, or spectral filtering in molecular electronics continues to be a central yet elusive goal in the field.^19–23^ Published works have elucidated those resonances and antiresonances can be achieved experimentally in molecular-scale junctions through gating^24–27^ and electrochemical^28,29^ methods. Experimental STM research by Nichols’s group has shown that Fano resonances can significantly modulate electrical conductance in molecular systems. For instance, conductance is enhanced when a Fano resonance is present in a charge transfer complex formed between a molecular backbone and tetracyanoethylene (TCNE).^30–33^ In contrast, conductance is suppressed by a Fano antiresonance, which results from the hybridization of localized metal d-orbitals with delocalized π-ligands, as demonstrated in ferrocene-based systems.^34–36^

The emergence of a Fano resonance in a molecular-scale junction hinges on the coexistence of a broad continuum energy spectrum and a well-defined, discrete bound state. A fundamental requirement is that the characteristic energy of this bound state remains stable and is not significantly broadened or shifted by the molecule-metal coupling interaction.^37^ The primary objective of this study is to systematically investigate the influence of molecular pendant groups (PGs) on the electric conductance of a series of bithiophene-bridged molecular wires. All three target molecules are terminated with thiol anchor groups to facilitate binding to gold electrodes, as illustrated in Figure 1.

**Figure 1:**
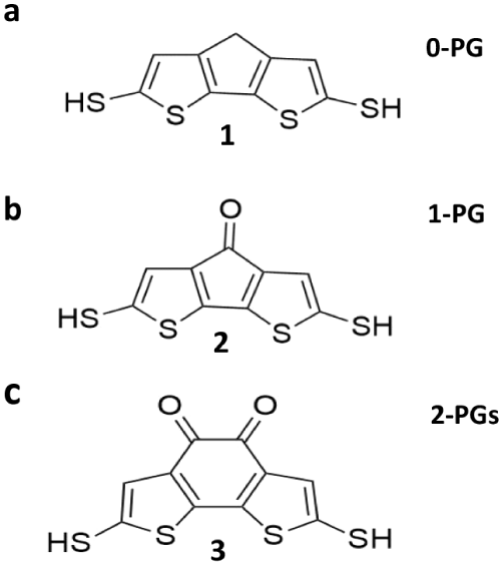
Schematic illustrations of bithiophene-based molecular wires with terminal thiol (−SH) anchor groups. (a) Unmodified bridge. (b) Bridge functionalized with a single pendant group. (c) Bridge functionalized with a two pendant groups.

## 2 Methods

To model electrical transport in bithiophene-based molecular wires with thiol anchor groups, we first optimized the S–Au binding geometry. Each compound was relaxed in the presence of fixed gold leads until atomic forces were below 0.05 eV/Å, using the SIESTA^38^ density functional code. Our calculations employed a double-ζ plus polarization basis set, norm-conserving pseudopotentials, and the LDA exchange-correlation functional, with a real-space grid defined by a 250 Ry energy cutoff. Tests with GGA produced comparable transmission functions. The Au leads were modeled to mimic break-junction conditions, consisting of 6-layer Au(111) surfaces with 30 atoms per layer, terminated by a gold pyramid. After relaxing the molecular junctions in various orientations, the electrical conductance was computed (for more detail see^13,17,39^ ). To elucidate how quantum interference and electronic coupling can be precisely tuned by the strategic embedding of functional PGs, we first establish a baseline with a reference structure. This initial molecule consists of a bithiophene bridge with no pendant group, which we designate as 0-PG (Figure 1a).

This simple structure allows us to characterize the intrinsic conductive properties of the unmodified oligothiophene backbone. Subsequently, we introduce a single oxygen atom as a pendant group, attached directly to the central thiophene ring of the bridge. This modified structure, designated as 1-PG (Figure 1b), is engineered to probe the specific electronic effects of a single electron-donating substituent. It is important to note that while the central ring now bears the PG, the overall bridge in both 1a and 1b consists of three five-membered rings. To further explore the structure–property relationship, a second pendant group is incorporated. This required a strategic modification to the molecular scaffold: the central ring was altered from a five-membered thiophene to a six-membered aromatic ring. This architectural change provides the necessary chemical space and geometric flexibility to accommodate a second oxygen atom without introducing excessive steric strain. The resulting molecule, featuring two pendant oxygen atoms on a modified central ring, is designated as 2-PG (Figure 1c). This comparative approach across the series (0-PG, 1-PG, and 2-PG) enables a detailed analysis of how the number and structural context of pendant groups modulate electron transport through the molecular junction.

## 3 Results and Discussion

In the present work, we define a “silent” Fano resonance as a quantum interference feature that is intrinsically present in the coherent electron transmission spectrum *T*(*E*), for a single, fixed junction conformation, but is rendered unobservable in the experimentally measured conductance. This extrinsic suppression is specifically attributed to the ensemble averaging over stochastic geometric fluctuations of the molecular junction, which smears the distinct spectral line shape, rather than to an intrinsic cancellation within the electronic structure of a static configuration. This study modeled the electronic structure and charge transport properties of three bithiophene-bridged derivatives, each functionalized with two terminal thiol anchor groups. As an initial step to understand their electronic characteristics, the frontier molecular orbitals (FMOs) specifically the highest occupied molecular orbital (HOMO) and the lowest unoccupied molecular orbital (LUMO) of derivatives **1–3** were computed. The spatial distribution of these FMOs, illustrated in Figure 2, provides critical insight into their conductive behavior. The analysis was guided by the orbital product rule (OPR), a principle first reported by,^40^ which establishes a key relationship between the FMOs and the efficiency of charge transport. This rule posits a linear relationship between molecular conductance and the amplitude of the relevant FMO at the molecular junctions. Specifically, to achieve high electrical conductance, the electron density of the frontier orbital mediating the charge transfer must be significantly delocalized and exhibit large amplitudes at both the entry and exit points of the molecule, which are defined by the thiol anchor groups. Conversely, low conductance is predicted when the orbital amplitude at either anchor is small. The Orbital Product Rule, which states that the transmission near the Fermi energy scales with the product of the coupling strengths |Γ_L_Γ_R_| of the frontier orbital to the left and right electrodes, provides a robust qualitative guide for trends within a homologous series of molecular junctions under coherent transport. Its applicability assumes that off-resonant tunneling through a single dominant orbital channel governs the conductance, and it does not account for strong interference effects or significant contributions from multiple orbitals near *E*_F_.

**Figure 2:**
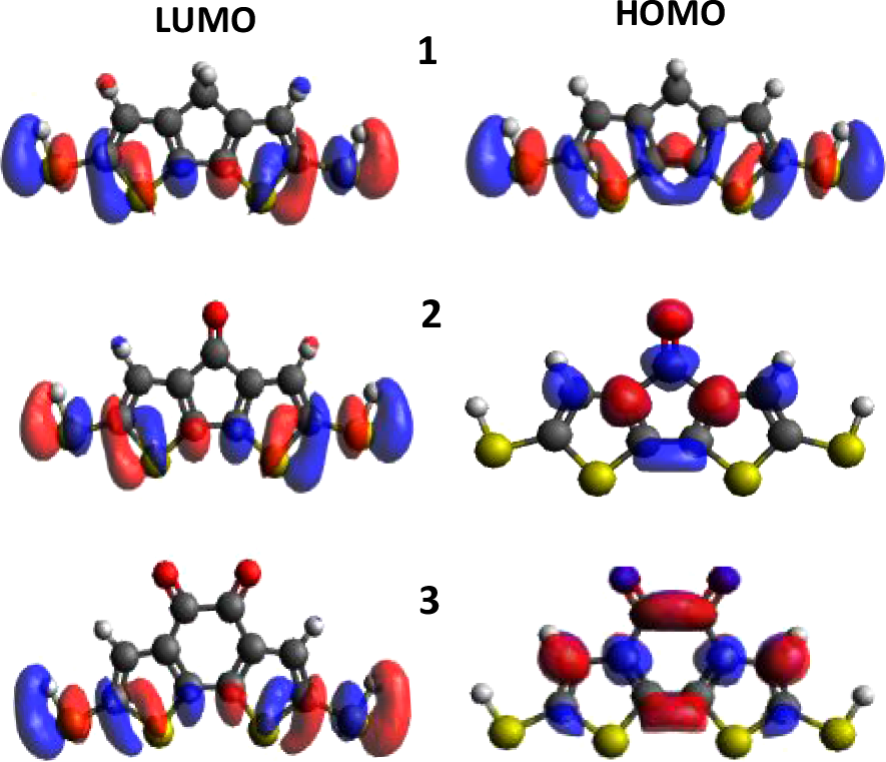
Spatial distributions of the frontier molecular orbitals (HOMO and LUMO) for molecules **1–3**. A key observation is the pronounced electron amplitude on the thiol anchors in the LUMOs across all molecules. In contrast, the HOMOs of molecules **2** and **3** exhibit no significant amplitude on these anchor groups, suggesting a potential asymmetry in charge injection efficiency.

The analysis of the Frontier Molecular Orbitals (FMOs) presented in Figure 2 provides a clear rationale for the predicted electrical conductance (*G*) of the three molecular systems (**1**, **2**, and **3**). The HOMO (Highest Occupied Molecular Orbital) level, which is the primary pathway for charge transport in these systems, exhibits distinct regions of positive (red) and negative (blue) phase, as well as significant variations in orbital amplitude. Critically, the amplitude of the orbital on the molecular anchors—the thiol groups that connect the molecule to the electrodes—is the determining factor for conductance. In molecule **1**, the HOMO features a high orbital amplitude directly on its anchor points. This indicates a strong electronic coupling between the molecule and the electrodes, facilitating efficient electron transport and resulting in a predicted large electrical conductance. In stark contrast, the HOMO orbitals of molecules **2** and **3** show little to no amplitude (low or zero weight) on their respective thiol anchors. This absence of electron density at the critical interface between the molecule and the electrode creates a weak electronic coupling. Consequently, based on the orbital product rule (OPR), the charge transport pathway is effectively broken, leading to the prediction of a small electrical conductance for both molecules **2** and **3**.

Following the discussion on OPR conductance predictions, a detailed investigation into the transport properties of a series of three bithiophene-bridged molecular derivatives was conducted. This study employed a robust computational framework combining density functional theory (DFT) with the nonequilibrium Green’s function (NEGF) formalism to model the quantum transport phenomena. The core of the analysis centered on calculating the electronic transmission function, *T*(*E*), which quantifies the probability of an electron with a specific energy *E* being transmitted coherently from the source electrode to the drain electrode through the molecular junction. By integrating this energy-dependent transmission coefficient around the Fermi level at a finite bias, the electrical conductance (*G*) for each derivative was precisely determined, providing a quantitative prediction of their performance as molecular-scale circuit elements at room temperature.

Figure 3 presents the density functional theory (DFT) calculated transmission spectra for the molecular derivatives **1–3** (structures shown in Figure 1). A clear correlation is observed between the molecular orbital characteristics depicted in Figure 2 and their corresponding charge transport properties. Derivative **1** (orange curve) exhibits the highest electronic conductance near the midgap region $\left(E-E_{F}^{\mathrm{DFT}}\approx 1.3\;\mathrm{eV}\right)$ , as indicated by the vertical dashed line. This enhanced conductance is a direct consequence of its favorable orbital anchoring: both the highest occupied (HOMO) and lowest unoccupied (LUMO) molecular orbitals possess a significant amplitude on the terminal thiol groups, which facilitates strong electronic coupling to the gold electrodes and efficient charge transport through the molecule. In contrast, derivatives **2** and **3** (purple and green curves, respectively) show markedly lower conductance values at the same midgap energy. This suppression in conductance is attributed to their orbital structures, both molecules feature precisely zero amplitude on their anchor groups for the frontier orbitals (HOMO and LUMO). This ineffective coupling to the electrodes creates a large tunneling barrier, resulting in poor conductance. The energy alignment reference for this analysis is the DFT-estimated Fermi energy $\left(E_{F}^{\mathrm{DFT}}\right)$ , which is set to coincide with the middle of the HOMO–LUMO gap. This convention is well-justified, as prior experimental-theoretical comparisons^41–44^ for poly aromatic hydrocarbons have consistently demonstrated that the dominant electron transport pathway occurs near the center of the HOMO–LUMO energy gap.

**Figure 3:**
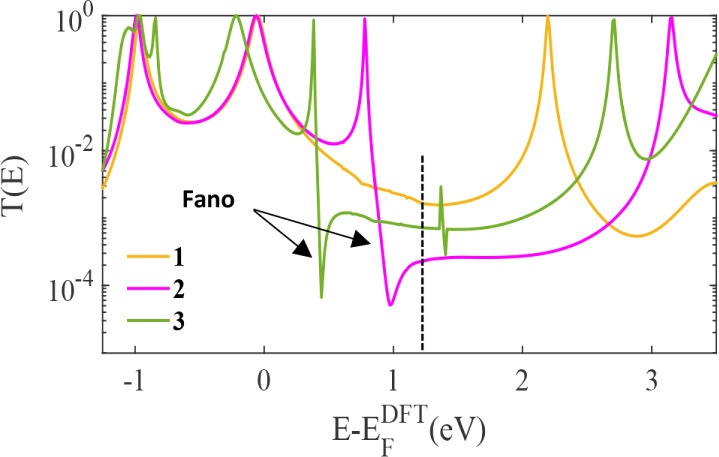
Zero bias transmission coefficients *T­(E),* obtained from density functional theory (DFT), for three different bithiophenes bridged derivatives in Au–Au junctions (Figure 1a–c). No pendant-group bridge **1** (orange-line), one pendant-group bridge **2** (purple-line), and two pendant-groups bridge **3** (green-line). **2** and **3** exhibit one and two Fano resonances, respectively.

Examining the transmission function spectrum, a distinct Fano resonance is observed in the HOMO–LUMO gap, more precisely around $E-E_{F}^{\mathrm{DFT}}$ = 1 eV for the system represented by the purple curve (**2**). A similar, though slightly shifted, resonant feature appears at approximately $E-E_{F}^{\mathrm{DFT}}$ = 0.5 eV for the green curve (**3**). The emergence of such asymmetric Fano line shapes in molecular junctions is a quantum interference effect that requires the coexistence of two distinct types of electronic pathways: a broad continuum of states and a narrow discrete state.

The necessary conditions are typically met by the hybridization of two different types of frontier molecular orbitals (FMOs). The first is a delocalized orbital, often from the molecular backbone, which provides a wide band of energy levels for electron transmission. The second is a localized orbital, frequently associated with a pendant side group, which creates a sharp, discrete energy state.^45,46^ This theoretical framework is well-supported by numerous studies,^47–54^ which demonstrate that the constructive and destructive interference between the electrons traversing the delocalized (continuum) pathway and those tunneling through the localized (discrete) state gives rise to the characteristic Fano resonance line shape observed in the conductance.

The schematic in Figure 4a illustrates the fundamental principle behind creating a Fano resonance: the quantum interference between a discrete, localized state and a broad, continuous spectrum. This concept is realized molecularly in Figure 4b. Derivative **1**, with both its HOMO and LUMO delocalized, provides only a continuous conduction path. In contrast, derivative **2** has a localized HOMO (on the carbazole) and a delocalized LUMO, establishing the two interfering pathways. The consequence is shown in Figure 4c, where the interaction between these pathways in derivative **2** generates a sharp, asymmetric Fano line shape in the transmission function. This definitive spectral feature is direct evidence of quantum interference and the successful design of a Fano resonance.

**Figure 4:**
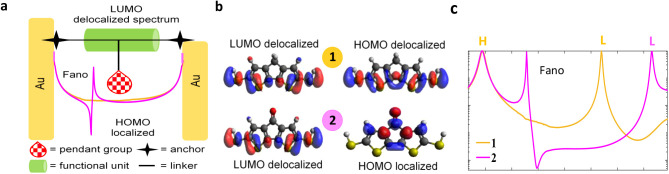
Schematic illustration of Fano resonance molecular design and DFT simulations. (a) Representation of forming a Fano resonance, the localized frontier molecular orbital embedded on the pending group in the molecular backbone can interact with the delocalized molecular orbital, thus shaping a Fano resonance as illustrated in tight binding model (TBM), calculations. (b) LUMO and HOMO frontier molecular orbitals of **1** and **2**. Oxygen atom is considered to be the pending group in **2**, this results in a localized HOMO embedded in the central core (carbazole), as the discrete spectrum, whereas the LUMO is delocalized and distributed along the molecular backbone as the continuum spectrum (bottom panel). For **1**, both LUMO and HOMO are delocalized and distributed along the molecular backbone as the continuous spectrum (top panel). (c) DFT transmission function *T­(E),* exhibit the Fano line shape resonance in the HOMO–LUMO gap of derivative **2** (purple curve), in addition to a smooth orange curve for derivative **1**.

Our theoretical demonstration of Fano resonance, arising from the quantum interference between localized and delocalized frontier molecular orbitals (FMOs) (Figure 4), aligns well with a recent experimental study that utilized an electrochemically gated scanning tunneling microscope break junction (EC-STM-BJ) technique.^55^ That work successfully identified a single Fano resonance in a carbazole core system featuring a nitrogen atom as a pendant group. Building upon this foundation, we now expand the research frontier to a more complex system: a carbazole core engineered with two pending oxygen atoms embedded in its central six-membered ring (Figure 1c). This “two-Fano” system introduces a second interference pathway, allowing us to probe the interplay of multiple resonances and their collective effect on quantum transport.

The introduction of a second pending oxygen atom in derivative **3** unequivocally induces a Fano resonance, as is clearly evidenced by the distinct asymmetric line shape observed in the green curve of Figure 3 at approximately $E-E_{F}^{\mathrm{DFT}}$ = 0.5 eV. This feature is a direct manifestation of the new quantum interference pathway created by the additional atom. However, assigning this resonance exclusively to either the left or right oxygen atom is not straightforward. The electronic structure of the molecule must be treated as a coherent quantum system, the observed Fano resonance is an emergent property of the entire molecular junction, where the interference is sculpted by the precise spatial arrangement and coupling of both oxygen atoms to the molecular backbone and electrodes.

Furthermore, a closer examination reveals a more subtle feature near $E-E_{F}^{\mathrm{DFT}}$ = 1.5 eV. Its significantly attenuated magnitude, especially when compared to the robust Fanos in the green and purple curves, suggests it represents a suppressed quantum interference effect. This observation aligns compellingly with the concept of Silent Frontier Molecular Orbitals (SFMOs), as discussed in reference.^20^ The SFMO effect posits that certain molecular orbitals, often due to weak or asymmetrical coupling to the electrodes, can fail to contribute significantly to the net conductance. Consequently, the quantum interference features associated with these “silent” orbitals are quenched and underrepresented in the transport spectrum. The faint signature at 1.5 eV is a prime candidate for such a suppressed resonance, indicating the presence of an interference pathway that is electronically disfavored and thus does not develop into a full, pronounced Fano line shape. The electronic distribution in derivative **3** reveals a clear asymmetry between its frontier orbitals. As shown in Figure 2, the HOMO is intensely localized on the electron-rich carbazole core, whereas the LUMO exhibits broad delocalization along the molecular framework. This localization has a crucial implication for quantum transport. The Fano resonance linked to the HOMO is highly sensitive to the precise electronic coupling at the molecule-electrode interfaces. In a real-world experimental setting, the binding configuration of the terminal thiol groups to the gold electrodes is never static; it is subject to tiny, continuous fluctuations. These nanoscale variations disrupt the delicate quantum interference necessary for the Fano resonance, effectively quenching it. Figure 5 provides direct evidence of this phenomenon, plotting *T*(*E*) for two nearly identical binding configurations. The stark contrast between the two curves underscores how minor geometric fluctuations can obliterate the Fano resonance, leaving only the more robust HOMO-based transport channel.

**Figure 5:**
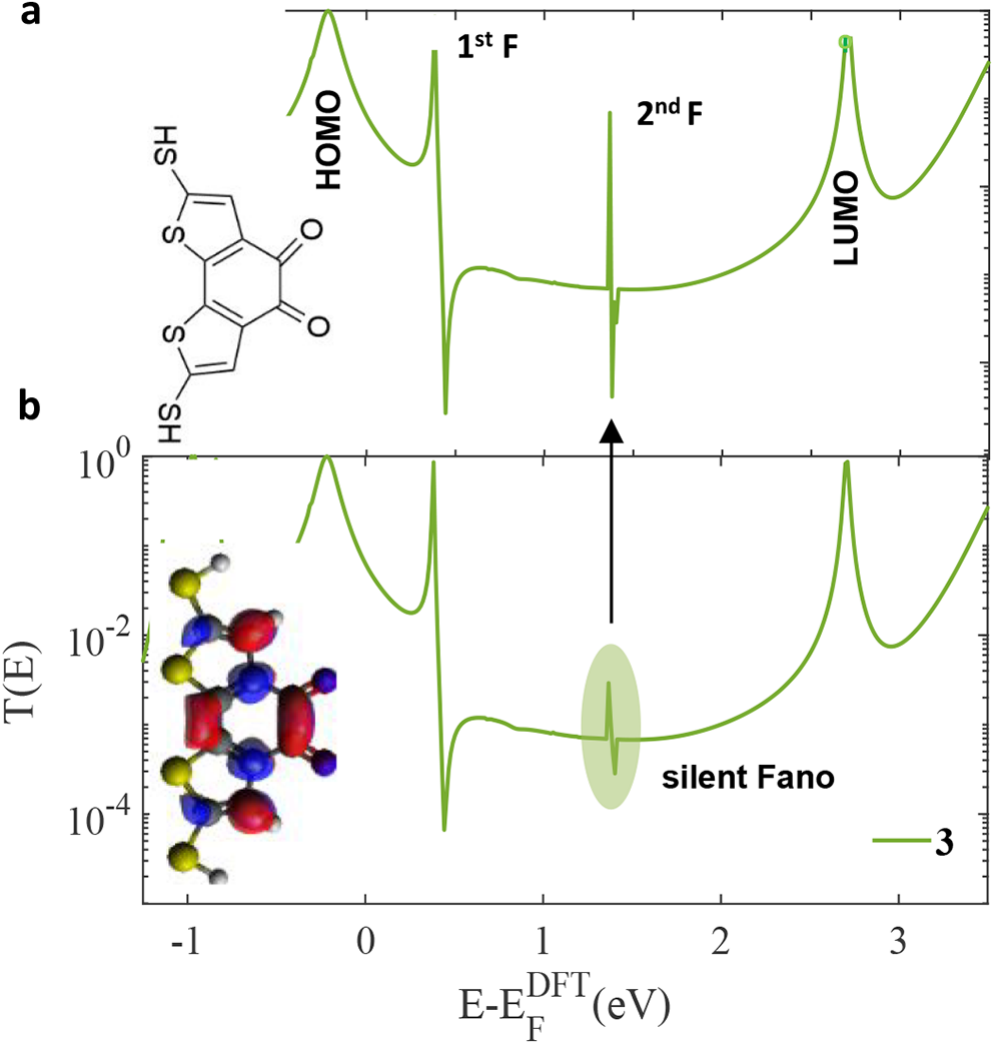
Transmission functions of the bithiophenes bridged derivative **3**. The top green (a), shows the transmission function of slightly asymmetric junction, for which the transmission Fano resonance at energy *E* = 1.5 eV, falls nonsilent. The bottom green curve (b), corresponds to an improbable symmetric situation, in which each thiol terminal group binds with precisely the same geometry to atomically identical electrodes.

The bottom transmission curve in Figure 5 corresponds to the theoretically intriguing yet experimentally improbable case of a perfectly symmetric molecular junction. In this idealized scenario, each terminal group binds to the electrodes with precisely identical geometry, creating a mirror-image symmetry across the molecular bridge. This high degree of spatial symmetry has a profound and direct consequence on the electronic transmission, giving rise to two distinct resonant features. A narrow Fano line shape resonance is present at the energy of HOMO (*E*_H_), while a much broader Breit–Wigner resonance appears at the energy of LUMO (*E*_L_). Critically, the transmission probability *T*(*E*) reaches the theoretical maximum of unity at the peak of both resonances *T*(*E*_H_) = 1 and *T*(*E*_L_) = 1indicating perfectly efficient electron transport through these orbital channels. These unitary transmission values are not a coincidence but are a direct quantum-mechanical consequence of the junction’s spatial symmetry. This symmetry enforces a specific coupling condition between the molecular orbitals and the electronic states of the electrodes, allowing for complete destructive interference in the electron paths around the resonances and resulting in maximal conduction.^56,57^ This study provides a blueprint for designing molecular quantum devices, highlighting that achieving predicted functions requires simultaneous optimization of both the molecule’s electronic structure and the junction geometry. A detailed comparison of the zero-bias transmission coefficients, *T*(*E*) for the molecular bridges with 2-PGs and without (0-PG) pendant groups is provided in Section 4 of the Supporting Information. This analysis confirms that the pendant groups introduce localized states, which interact with the conduction continuum to produce sharp Fano resonances, fundamentally altering the electron transport pathway.

The “silent” Fano resonances identified here are distinct from standard destructive quantum interference (DQI) antiresonances. A conventional DQI feature manifests as a pronounced dip or near-zero transmission at a specific energy within the measured spectrum of a static junction, arising from the intrinsic phase cancellation of electron waves. In contrast, a “silent” resonance is a theoretically predicted, sharp Fano line-shape (with both peak and dip features) for a single static geometry. Its “silence” that is, its absence from the observable, time-averaged spectrum is not due to an intrinsic cancellation, but to the extrinsic smearing of its sharp profile through ensemble averaging over thermally accessible geometrical fluctuations. This mechanism highlights how junction dynamics can selectively obscure coherent interference phenomena without eliminating the underlying electronic states.

## 4 Conclusions

In conclusion, this study establishes a comprehensive theoretical framework for understanding and manipulating quantum interference (QI) in bithiophene-bridged molecular junctions. We have quantitatively confirmed that the orbital product rule governs the baseline conductance, while the strategic introduction of pendant atoms enables the precise engineering of Fano resonances within the transmission spectrum. Crucially, our findings reveal that the measurable conductance is not solely an intrinsic molecular property but is profoundly shaped by the molecule-electrode interface. The extreme sensitivity of certain QI features to minute structural fluctuations explains their “silent” character in experiments and underscores the critical challenge of junction stability. Ultimately, this work provides a detailed blueprint for designing molecular-scale quantum interference devices, highlighting that achieving predicted functionalities requires simultaneous optimization of both molecular electronic structure and junction geometry. Furthermore, this work opens new ideas for designing new nanotechnology devices with potential practical applications.

## Supplementary Material



## Notes

Optimized geometries of bithiophene-based molecular wires were obtained using the SIESTA package with a double-ζ polarized (DZP) basis set and a 300 Ry mesh cutoff; generalized gradient approximation (GGA) was employed for exchange-correlation, relaxing structures until all forces were below 0.02 eV/Å (PDF)

## References

[1] Su T. A., Neupane M., Steigerwald M. L., Venkataraman L., Nuckolls C. (2016). Chemical principles of single-molecule electronics. Nat. Rev. Mater..

[2] Aradhya S. V., Venkataraman L. (2013). Single-molecule
junctions beyond electronic transport. Nat.
Nanotechnol..

[3] Whalley A. C., Steigerwald M. L., Guo X., Nuckolls C. (2007). Reversible switching
in molecular electronic devices. J. Am. Chem.
Soc..

[4] Dowling J. P., Milburn G. J. (2003). Quantum technology: the second quantum revolution. Proc. R. Soc. London, Ser. A.

[5] Ohshita J., Nodono M., Kai H., Watanabe T., Kunai A., Komaguchi K., Shiotani M., Adachi A., Okita K., Harima Y. (1999). Synthesis and optical, electrochemical, and electron-transporting
properties of silicon-bridged bithiophenes. Organometallics.

[6] Ye J., Al-Jobory A., Zhang Q.-C., Cao W., Alshehab A., Qu K., Alotaibi T., Chen H., Liu J., Ismael A. K. (2022). Highly insulating alkane rings with destructive σ-interference. Sci. China Chem..

[7] Cui X., Primak A., Zarate X., Tomfohr J., Sankey O., Moore A., Moore T., Gust D., Harris G., Lindsay S. (2001). Reproducible measurement of single-molecule conductivity. Science.

[8] Wang X., Ismael A., Ning S., Althobaiti H., Al-Jobory A., Girovsky J., Astier H. P. A. G., O’Driscoll L. J., Bryce M. R., Lambert C. J. (2022). Electrostatic
Fermi level tuning in large-scale self-assembled monolayers of oligo­(phenylene–ethynylene)
derivatives. Nanoscale Horiz..

[9] Xu B., Tao N. J. (2003). Measurement of single-molecule
resistance by repeated
formation of molecular junctions. Science.

[10] O’brien J. L., Furusawa A., Vučković J. (2009). Photonic quantum technologies. Nat. Photonics.

[11] Seidl F. (1949). An Interpretation
of the Resonance Scattering of Neutrons by Cobalt in Terms of the
Breit-Wigner Equation. Phys. Rev..

[12] Fano U. (1961). Effects of
Configuration Interaction on Intensities and Phase Shifts. Phys. Rev..

[13] Ismael A. K., Grace I., Lambert C. J. (2015). Increasing
the thermopower of crown-ether-bridged
anthraquinones. Nanoscale.

[14] van
Beveren E., Rupp G. (2001). Modified Breit–Wigner formula
for mesonic resonances describing OZI decays of confined states and
the light scalar mesons. Eur. Phys. J. C.

[15] Guo W.-L., Wu Y.-L. (2009). Enhancement
of dark matter annihilation via Breit-Wigner resonance. Phys. Rev. D:Part., Fields, Gravitation, Cosmol..

[16] Miroshnichenko A. E., Flach S., Kivshar Y. S. (2010). Fano resonances
in nanoscale structures. Rev. Mod. Phys..

[17] Ismael A. K., Al-Jobory A., Grace I., Lambert C. J. (2017). Discriminating single-molecule
sensing by crown-ether-based molecular junctions. J. Chem. Phys..

[18] Passarelli N., Pérez L. A., Coronado E. A. (2014). Plasmonic interactions: from molecular
plasmonics and fano resonances to ferroplasmons. ACS Nano.

[19] Norimoto S., Nakamura S., Okazaki Y., Arakawa T., Asano K., Onomitsu K., Kobayashi K., Kaneko N.-H. (2018). Fano effect in the
transport of an artificial molecule. Phys. Rev.
B.

[20] Ismael A. K., Lambert C. J. (2020). Molecular-scale thermoelectricity: a worst-case scenario. Nanoscale Horiz..

[21] Molle A., Dubois A., Gorfinkiel J. D., Cederbaum L. S., Sisourat N. (2021). Fano interferences in environment-enabled
electron
capture. Phys. Rev. A.

[22] Alshammari M., Al-Jobory A. A., Alotaibi T., Lambert C. J., Ismael A. (2022). Orientational
control of molecular scale thermoelectricity. Nanoscale Adv..

[23] Limonov M. F., Rybin M. V., Poddubny A. N., Kivshar Y. S. (2017). Fano resonances
in photonics. Nat. Photonics.

[24] Capozzi B., Xia J., Adak O., Dell E. J., Liu Z.-F., Taylor J. C., Neaton J. B., Campos L. M., Venkataraman L. J. N.
N. (2015). Single-molecule
diodes with high rectification ratios through environmental control. Nat. Nanotechnol..

[25] Wang X., Ismael A., Ning S., Althobaiti H., Al-Jobory A., Girovsky J., Astier H. P. A. G., O’Driscoll L. J., Bryce M. R., Lambert C. J. (2022). Electrostatic
Fermi level tuning in large-scale self-assembled monolayers of oligo
(phenylene–ethynylene) derivatives. Nanoscale
Horiz..

[26] Alanazi B., Alajmi A., Aljobory A., Lambert C., Ismael A. (2024). Tuning quantum
interference through molecular junctions formed from cross-linked
OPE-3 dimers. J. Mater. Chem. C.

[27] Ratner M.
A. (2002). Introducing
molecular electronics. Materials Today.

[28] Li Y., Buerkle M., Li G., Rostamian A., Wang H., Wang Z., Bowler D. R., Miyazaki T., Xiang L., Asai Y. (2019). Gate controlling
of
quantum interference and direct observation of anti-resonances in
single molecule charge transport. Nat. Mater..

[29] Huang B., Liu X., Yuan Y., Hong Z.-W., Zheng J.-F., Pei L.-Q., Shao Y., Li J.-F., Zhou X.-S., Chen J.-Z. (2018). Controlling
and observing sharp-valleyed quantum interference effect
in single molecular junctions. J. Am. Chem.
Soc..

[30] Wang K., Vezzoli A., Grace I. M., McLaughlin M., Nichols R. J., Xu B., Lambert C. J., Higgins S. J. (2019). Charge
transfer complexation boosts molecular conductance through Fermi level
pinning. Chem. Sci..

[31] Vezzoli A., Grace I., Brooke C., Wang K., Lambert C. J., Xu B., Nichols R. J., Higgins S. J. (2015). Gating of single molecule junction
conductance by charge transfer complex formation. Nanoscale.

[32] Naghibi S., Ismael A. K., Vezzoli A., Al-Khaykanee M. K., Zheng X., Grace I. M., Bethell D., Higgins S. J., Lambert C. J., Nichols R. J. (2019). Synthetic Control of Quantum Interference
by Regulating Charge on a Single Atom in Heteroaromatic Molecular
Junctions. J. Phys. Chem. Lett..

[33] Ismael A. K. (2023). 20-State
Molecular Switch in a Li@ C60 Complex. ACS Omega.

[34] Pan H., Yang P., Wang Y., Li J., Li S., Hou S. (2023). A designing
strategy for fano resonances in molecular junctions. J. Phys. Chem. C.

[35] Alotaibi T., Alshahrani M., Alshammari M., Alotaibi M., Taha T. A. M., Al-Jobory A. A., Ismael A. (2024). Orientational Effects and Molecular-Scale
Thermoelectricity Control. ACS Omega.

[36] Bai J., Daaoub A., Sangtarash S., Li X., Tang Y., Zou Q., Sadeghi H., Liu S., Huang X., Tan Z. (2019). Anti-resonance features of destructive quantum interference in single-molecule
thiophene junctions achieved by electrochemical gating. Nat. Mater..

[37] Lambert C. J. (2015). Basic concepts
of quantum interference and electron transport in single-molecule
electronics. Chem. Soc. Rev..

[38] Soler J. M., Artacho E., Gale J. D., García A., Junquera J., Ordejón P., Sánchez-Portal D. (2002). The SIESTA
method for ab initio order-N materials simulation. J. Phys.:Condens. Matter.

[39] Al-Jobory A. A., Ismael A. K. (2023). Controlling quantum interference
in tetraphenyl-aza-BODIPYs. Curr. Appl. Phys..

[40] Lambert C. J., Liu S.-X. (2018). A magic ratio rule
for beginners: a chemist’s
guide to quantum interference in molecules. Chem. - Eur. J..

[41] Xiao C., Li Z., Li K., Huang P., Xie Y. (2014). Decoupling interrelated
parameters for designing high performance thermoelectric materials. Acc. Chem. Res..

[42] Liu S.-X., Ismael A. K., Al-Jobory A., Lambert C. J. (2023). Signatures of Room-Temperature
Quantum Interference in Molecular Junctions. Acc. Chem. Res..

[43] Wang X., Ismael A., Almutlg A., Alshammari M., Al-Jobory A., Alshehab A., Bennett T. L., Wilkinson L. A., Cohen L. F., Long N. J. (2021). Optimised power harvesting by controlling
the pressure applied to molecular junctions. Chem. Sci..

[44] Ismael A., Wang X., Bennett T. L., Wilkinson L. A., Robinson B. J., Long N. J., Cohen L. F., Lambert C. J. (2020). Tuning
the thermoelectrical properties of anthracene-based self-assembled
monolayers. Chem. Sci..

[45] Stadler R., Markussen T. (2011). Controlling
the transmission line shape of molecular
t-stubs and potential thermoelectric applications. J. Chem. Phys..

[46] Ismael A.
K., Mohaymen
Taha T. A., Al-Jobory A. (2024). Three distinct conductance states
in polycyclic aromatic hydrocarbon derivatives. RR. Soc. Open Sci..

[47] Papadopoulos T., Grace I., Lambert C. (2006). Control of electron transport through
Fano resonances in molecular wires. Phys. Rev.
B.

[48] Ismael A. K., Grace I., Lambert C. J. (2017). Connectivity dependence of Fano resonances
in single molecules. Phys. Chem. Chem. Phys..

[49] Ismael A.
K., Lambert C. J. (2019). Single-molecule
conductance oscillations in alkane
rings. J. Mater. Chem. C.

[50] Tada T., Yoshizawa K. (2002). Quantum transport
effects in nanosized graphite sheets. Chemphyschem.

[51] Tada T., Yoshizawa K. (2003). Quantum transport effects in nanosized graphite sheets.
II. Enhanced transport effects by heteroatoms. J. Phys. Chem. B.

[52] Tada T., Yoshizawa K. (2004). Reverse exponential
decay of electrical transmission
in nanosized graphite sheets. J. Phys. Chem.
B.

[53] Yoshizawa K., Tada T., Staykov A. (2008). Orbital views of the electron transport
in molecular devices. J. Am. Chem. Soc..

[54] Alshammari M., Alotaibi T., Alotaibi M., Ismael A. K. (2023). Influence of Charge
Transfer on Thermoelectric Properties of Endohedral Metallofullerene
(EMF) Complexes. Energies.

[55] Zheng Y., Duan P., Zhou Y., Li C., Zhou D., Wang Y., Chen L. C., Zhu Z., Li X., Bai J. (2022). Fano Resonance in Single-Molecule Junctions. Angew. Chem..

[56] Fukui, K. Theory of orientation and stereoselection. In Orientation and Stereoselection; Springer, 2006; pp. 1–85.

[57] Woodward R. B., Hoffmann R. (1970). The conservation of orbital symmetry. Angew. Chem., Int. Ed. Engl..

