# Supplementary material for: Beyond the Dip: Silent and Non-Silent Fano Resonances in Quantum Systems
Source: ACS Omega. 2026 Jan 16;11(4):6664–70. doi: 10.1021/acsomega.5c11827 (PMC12878757; doi:10.1021/acsomega.5c11827)
Supplement: Supplementary file 1 [file ao5c11827_si_001.pdf]

Beyond the Dip: Silent and Non-Silent Fano Resonances in Quantum Systems

Ali K. Ismael[a,b*]

[a] Physics Department, Lancaster University, Lancaster, LA1 4YB, UK

[b] Department of Physics, College of Education for Pure Science, Tikrit University, Tikrit, Iraq.

* Correspondence: k.ismael@lancaster.ac.uk

1. Optimised Density Functional Theory (DFT) structures of isolated molecular-scale structures.

The SIESTA[1-6] code was used to calculate the optimised geometries. The molecule depicted in **Error! Reference source not found.** (manuscript) was optimised by relaxing the structure to obtain the most stable geometry, which happens when all forces are less than $0.01 eV$. A real-space grid is defined by a double-polarized basis set (DZP) with a $250\ Ry$ equivalent energy cutoff. The generalised gradient approximation (GGA) is selected to be the exchange-correlation functional. Figure S) shows fully relaxed geometries of the studied molecule in three bithiophene-based forms.

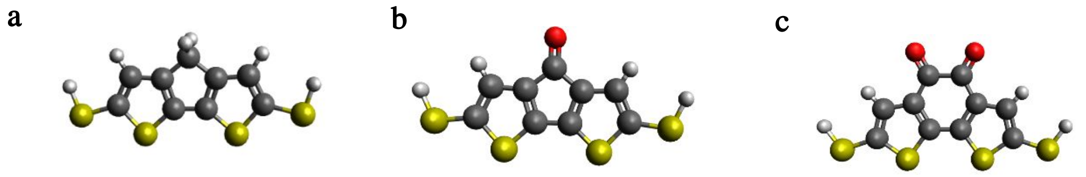

Figure S1: Fully relaxed calculated molecular structures of the bithiophene-based molecular wires with terminal thiol (-SH) anchor groups. a) unmodified bridge. b) bridge functionised with a single pendant group. c) bridge functionised with a two pendant groups.

2. Binding energy

This section presents the binding energy calculations for the four studied macrocycles, focusing on their interaction with Au leads through thiol anchoring groups. All

molecules form bonds between the terminal thiol groups and gold electrodes (Au). The density functional theory (DFT) method was employed in conjunction with the counterpoise correction to eliminate the basis set superposition error (BSSE). The binding energy ($B.E$) is computed as the difference between the total energy of the combined system (E_{AB}) and the sum of the energies of the individual components (E_A and E_B), each computed in the presence of ghost atoms [7,8 9]:

$$Binding\ Energy = E_{AB} - E_A - E_B$$

The calculated optimised distances and corresponding binding energy is presented in **Error! Reference source not found.** below.

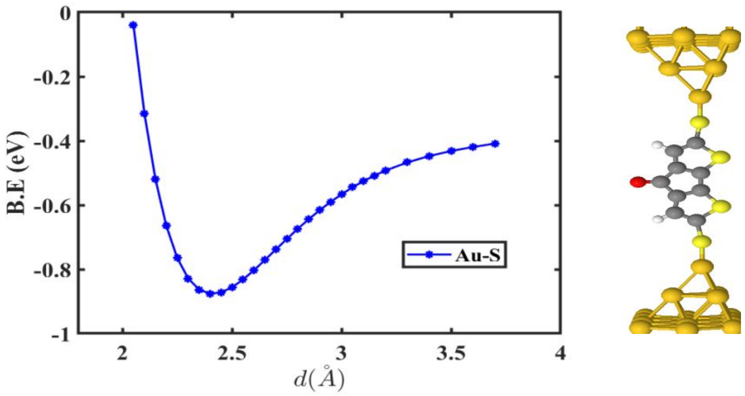

Figure S2: Left panel Binding configuration and energy curve for **2**, showing thiol anchoring to Au. Right panel. The optimized distance is 2.4 Å with a binding energy of $-0.88\ eV$.

3. Optimised DFT structures of compounds in their Junctions

Upon geometric optimization, the bithiophene-based molecules bind symmetrically to both Au electrodes in the simulated junctions. As illustrated in Figure S3, each terminal anchoring group adopts an identical bonding geometry on the atomically identical Au surfaces, resulting in structurally mirror-symmetric junctions. This computational design ensures the initial electronic coupling to the left and right electrodes is equivalent ($\Gamma_L \approx \Gamma_R$), a key condition for analysing the coherent transport properties of the isolated molecular backbone.

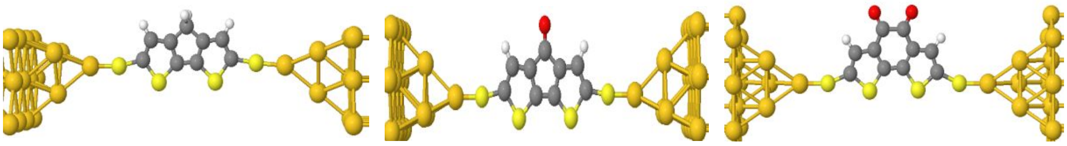

1 2 3

Figure S3: Optimised structures of **1-3** in Au|molecule|Au junctions. Junctions correspond to an improbable symmetric situation, in which each terminal group binds with precisely the same geometry to atomically identical electrodes.

The transmission functions presented in Figure 3 of the main text were calculated for the structurally symmetric junction geometries shown in Figure S3. In these configurations, the molecule binds with identical geometry to both electrodes, ensuring equivalent left and right electronic couplings ($\Gamma_L = \Gamma_R$).

In contrast, the transmission spectra in Figure 5 were obtained from a set of slightly asymmetric junction geometries. These structures were deliberately generated to model the effect of realistic electrode roughness and thermal fluctuations by introducing small, random variations in the bonding geometry at one electrode. This asymmetry breaks the perfect coupling symmetry ($\Gamma_L \neq \Gamma_R$), allowing us to investigate the stability of the transport features against junction disorder.

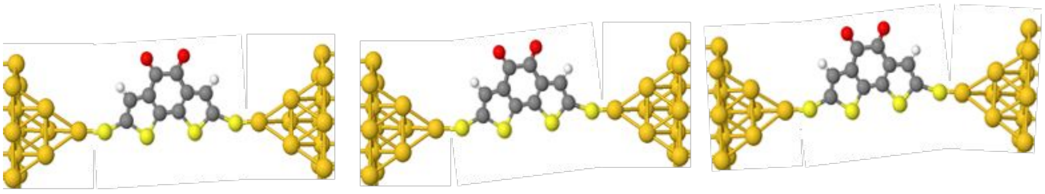

Figure S4: Optimised structures of **3** in Au|molecule|Au junctions. Junctions correspond to slightly asymmetric situations, in which each terminal group doesn't bind with precisely the same geometry to atomically identical electrodes.

4. Transmission coefficient comparison with and without pendant group

In this analysis, we compare the electron transmission characteristics of bithiophene-based molecular bridges in two distinct configurations: one with two pendant groups (2-PGs) and one without any pendant group (0-PG), as illustrated in Fig. S5. Fig. S6 presents the zero-bias transmission coefficients, T(E), calculated via density functional theory (DFT), for these molecular junctions between gold electrodes.

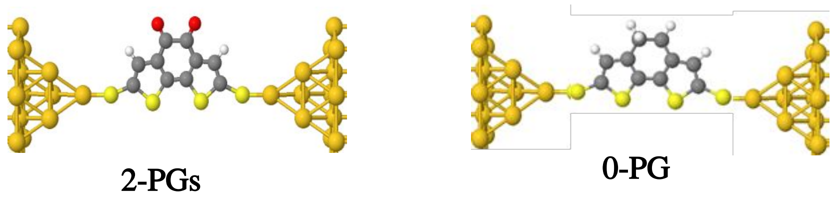

Figure S5: Optimised structures of bithiophene-based molecules with and without pendant group in Au|molecule|Au junctions. Left with two pendant groups (2-GPs), right without pendant group (0-GP).

The transmission spectrum for the 0-PG bridge (orange curve) exhibits a smooth, featureless profile, indicative of a typical coherent tunneling regime where electron transport occurs without significant interference or resonant scattering. This suggests a relatively direct coupling between the molecular backbone and the electrodes, with no localized states strongly interacting with the continuum. In stark contrast, the transmission spectrum for the 2-PGs bridge (green curve) reveals two distinct Fano resonance features. These asymmetric, sharp resonances signify quantum interference effects arising from the interaction between discrete localized states—introduced by the pendant groups—and the continuum of electronic states in the molecular backbone and electrodes. The presence of Fano resonances indicates that the pendant groups create localized molecular orbitals which couple to the conduction pathway, leading to constructive and destructive interference patterns that sharply modulate transmission at specific energies.

The comparison underscores a critical structure–function relationship: the introduction of pendant groups transforms the electron transport mechanism. While the bare bithiophene bridge (0-PG) acts as a simple tunneling barrier, the functionalized bridge (2-PGs) exhibits a more complex quantum-interference-dominated behavior. This has

significant implications for molecular electronics, as Fano resonances can be exploited for designing highly sensitive switches, sensors, or energy-filtering devices, where transmission can be drastically altered by small changes in energy, external fields, or molecular conformation. Thus, the DFT-based transmission analysis clearly demonstrates that pendant groups are not merely passive structural additions; they actively engineer the electronic transport landscape by introducing quantum interference effects, as evidenced by the emergence of Fano resonances in the 2-PGs system.

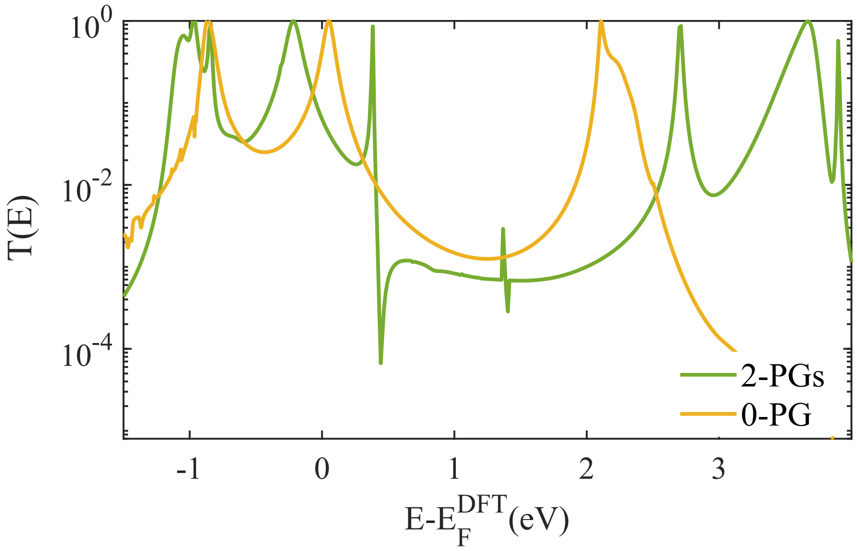

Figure S6: Zero bias transmission coefficients T(E), obtained from density functional theory (DFT), for two different bithiophenes bridged derivatives in Au-Au junctions (Fig. S5). Two pendant-group bridge 2-PGs (green-line), and no pendant-group bridge 0-PG (orang-line). 2-PGs exhibit two Fano resonances while 0-PG shows smooth curve (no Fano).

References

1. Kobko, N., & Dannenberg, J. J. (2001). Effect of Basis Set Superposition Error (BSSE) upon ab Initio Calculations of Organic Transition States. *The Journal of Physical Chemistry A*, *105*(10), 1944–1950.

2. Kohn, W., & Sham, L. J. (1965). Self-Consistent Equations Including Exchange and Correlation Effects. *Physical Review*, *140*(4A), A1133–A1138.

3. Perdew, J. P., Burke, K., & Ernzerhof, M. (1996). Generalized Gradient Approximation Made Simple. *Physical Review Letters*, *77*(18), 3865–3868.

4. Perdew, J. P., & Zunger, A. (1981). Self-interaction correction to density-functional approximations for many-electron systems. *Physical Review. B, Condensed Matter*, *23*(10), 5048–5079.

5. Sinnokrot, M. O., Valeev, E. F., & Sherrill, C. D. (2002). Estimates of the Ab Initio Limit for π−π Interactions: The Benzene Dimer. *Journal of the American Chemical Society*, *124*(36), 10887–10893.

6. Soler, J. M., Artacho, E., Gale, J. D., García, A., Junquera, J., Ordejón, P., & Sánchez-Portal, D. (2002). The SIESTA method forab initioorder-Nmaterials simulation. *Journal of Physics Condensed Matter*, *14*(11), 2745–2779.

7. Cao, Y., Huang, C., & Lu, Q. (2024). Photoelectrochemically driven iron-catalysed C(sp3)−H borylation of alkanes. *Nature Synthesis*, *3*(4), 537–544.

8. Ding, W. W., He, Z. Y., Sayed, M., Zhou, Y., Han, Z. Y., & Gong, L. Z. (2024). Enantioselective synthesis of β- and α-amino ketones through reversible alkane carbonylation. *Nature Synthesis*, *3*(4), 507–516.

9. Dragojlovic, V. Conformational analysis of cyclics. *ChemTexts* **1**, 14 (2015).
